# Supplementary material for: Optimization of sand casting performance parameters and missing data prediction
Source: R Soc Open Sci. 2019 Aug 7;6(8):181860. doi: 10.1098/rsos.181860 (PMC6731703; doi:10.1098/rsos.181860)
Supplement: Original data of foundry sand and MATLAB code of BP neural network [file rsos181860supp1.doc]

***Royal Society Open Science***

Optimization of sand casting performance parameters and missing data prediction

Qingwei Xu, Kaili Xu^*^, Li Li and Xiwen Yao^*^

Key Laboratory of Ministry of Education on Safe Mining of Deep Metal Mines, School of Resources and Civil Engineering, Northeastern University, Shenyang 110819, People's Republic of China

*Correspondence author: xklsafety@163.com (K.X), yxw_20061005@126.com (X.Y)

**Appendix A**

**Table S1. Original data of foundry sand**

| Batch No. | performance parameters | | | |
| --- | --- | --- | --- | --- |
|  | VQ (m^4^∙N^-1^∙S^-1^) | WCS (MPa) | MC (%) | Com (%) |
| 1 | 170 | 0.155 | 3.69 | 40 |
| 2 | 175 | 0.16 | 3.56 | 38 |
| 3 | 160 | 0.15 | 3.65 | 40 |
| 4 | 175 | 0.155 | 3.63 | 40 |
| 5 | 170 | 0.155 | 3.6 | 40.5 |
| 6 | 165 | 0.16 | 3.66 | 42 |
| 7 | 170 | 0.15 | 3.68 | 41 |
| 8 | 180 | 0.155 | 3.6 | 41.5 |
| 9 | 170 | 0.16 | 3.72 | 43 |
| 10 | 180 | 0.15 | 3.7 | 43 |
| 11 | 175 | 0.16 | 3.67 | 42 |
| 12 | 185 | 0.15 | 3.61 | 42.5 |
| 13 | 175 | 0.155 | 3.64 | 41 |
| 14 | 180 | 0.155 | 3.67 | 43 |
| 15 | 170 | 0.155 | 3.71 | 42 |
| 16 | 185 | 0.15 | 3.6 | 42 |
| 17 | 175 | 0.15 | 3.59 | 41.5 |
| 18 | 180 | 0.15 | 3.54 | 41 |
| 19 | 175 | 0.155 | 3.64 | 42 |
| 20 | 180 | 0.155 | 3.62 | 42 |
| 21 | 175 | 0.15 | 3.6 | 38 |
| 22 | 190 | 0.155 | 3.66 | 41.5 |
| 23 | 170 | 0.15 | 3.66 | 39.5 |
| 24 | 185 | 0.15 | 3.63 | 41 |
| 25 | 180 | 0.16 | 3.72 | 43 |
| 26 | 180 | 0.15 | 3.62 | 40.5 |
| 27 | 170 | 0.155 | 3.7 | 41.5 |
| 28 | 175 | 0.15 | 3.66 | 42.5 |
| 29 | 180 | 0.15 | 3.58 | 40 |
| 30 | 180 | 0.15 | 3.74 | 44 |
| 31 | 180 | 0.15 | 3.67 | 43 |
| 32 | 185 | 0.145 | 3.62 | 41 |
| 33 | 180 | 0.15 | 3.7 | 42 |
| 34 | 180 | 0.15 | 3.67 | 42 |
| 35 | 175 | 0.155 | 3.62 | 41.5 |
| 36 | 175 | 0.15 | 3.6 | 41.5 |
| 37 | 175 | 0.155 | 3.72 | 42.5 |
| 38 | 175 | 0.145 | 3.72 | 43 |
| 39 | 175 | 0.15 | 3.64 | 41 |
| 40 | 180 | 0.15 | 3.64 | 42 |

**Appendix B**

**MATLAB code of BP neural network in this paper**

data=xlsread('sample path');

input_train=data(1:39,2:4)';

output_train=data(1:39,1)';

[inputn,inputps]=mapminmax(input_train);

[outputn,outputps]=mapminmax(output_train);

net=newff(inputn,outputn,9,{'tansig','purelin'},'trainlm','learngd','mse');

net.trainParam.epochs=1000;

net.trainParam.goal=0.001;

net.trainparam.lr=0.001;

net=train(net,inputn,outputn);

prediction=sim(net,inputn);
